# Potential Use of Cannabinoids for the Treatment of Pancreatic Cancer

**DOI:** 10.1089/pancan.2018.0019

**Published:** 2019-01-25

**Authors:** Golnaz Sharafi, Hong He, Mehrdad Nikfarjam

**Affiliations:** Department of Surgery, University of Melbourne, Austin Health, Melbourne, Australia.

**Keywords:** cannabidiol, cannabinoids, pancreatic cancer, tetrahydrocannabinol

## Abstract

**Background:** Cannabinoid extracts may have anticancer properties, which can improve cancer treatment outcomes. The aim of this review is to determine the potentially utility of cannabinoids in the treatment of pancreatic cancer.

**Methods:** A literature review focused on the biological effects of cannabinoids in cancer treatment, with a focus on pancreatic cancer, was conducted. *In vitro* and *in vivo* studies that investigated the effects of cannabinoids in pancreatic cancer were identified and potential mechanisms of action were assessed.

**Results:** Cannabinol receptors have been identified in pancreatic cancer with several studies showing *in vitro* antiproliferative and proapoptotic effects. The main active substances found in cannabis plants are cannabidiol (CBD) and tetrahydrocannabinol (THC). There effects are predominately mediated through, but not limited to cannabinoid receptor-1, cannabinoid receptor-2, and G-protein-coupled receptor 55 pathways. *In vitro* studies consistently demonstrated tumor growth-inhibiting effects with CBD, THC, and synthetic derivatives. Synergistic treatment effects have been shown in two studies with the combination of CBD/synthetic cannabinoid receptor ligands and chemotherapy in xenograft and genetically modified spontaneous pancreatic cancer models. There are, however, no clinical studies to date showing treatment benefits in patients with pancreatic cancer.

**Conclusions:** Cannabinoids may be an effective adjunct for the treatment of pancreatic cancer. Data on the anticancer effectiveness of various cannabinoid formulations, treatment dosing, precise mode of action, and clinical studies are lacking.

## Introduction

Pancreatic cancer is the fourth major cause of cancer death and is likely to become the second major cause of cancer death after lung cancer by 2030, with an urgent need to improve treatment outcomes.^1,2^ There has been interest in the use of cannabinoids for the treatment of chemotherapy-related side effects such as cachexia, lethargy, and nausea, but their potential use as an anticancer agent is not well supported.^3,4^ Cannabinoids can be classified into plant-derived, endocannabinoids and synthetic groups.^5^

Plant-derived cannabinoids are largely the product of *Cannabis sativa* and *Cannabis indica* plant species.^6^ There are more than 60 phytocannabinoid entities found in cannabis plant extracts, among which there are currently two of main clinical interest: cannabidiol (CBD) and tetrahydrocannabinol (THC).^7,8^ Endocannabinoids are endogenous cannabinoids that include anandamide (AEA) and 2-arachidonoylglycerol (2-AG), involved in a variety of physiological and cognitive processes. Several synthetic cannabinoid compounds have been produced such as the aminoalkyl indole derivative WIN-55 that mimics THC and JWH-133, a potent CB2 receptor agonist that has anti-inflammatory effects. Pre-clinical studies have shown that certain synthetic cannabinoids also have antiangiogenic and antitumor effects.^5,9,10^

## Methods

A literature review of studies reporting on the cannabinoids in cancer treatment was undertaken with a focus on pancreatic cancer. An electronic search was conducted in PubMed, MEDLINE, EMBASE, and Web of Science for articles published from November 1961 until September 2018. References of relevant literature were reviewed to identify additional studies. *In vitro* and *in vivo* studies that have investigated the effects of cannabinoids in pancreatic cancer, including potential mechanisms of action and signaling pathways, were assessed.

## Results and Discussion

### Cannabis plants

Cannabinoids are compounds extracted from two types of plants, *C. sativa* and *C. indica*. Cannabis plants contain more than 400 chemical compounds, over 60 of them are phytocannabinoid entities, including delta-8-THC (d8-THC), cannabinol (CBN), cannabicyclol (CBL), cannabichromene (CBC), and cannabigerol (CBG);although THC and CBD are recognized as the two main substances, THC, CBD, and CBN have mostly been used in different studies.^8,11–13^ These plants can be differentiated by their physical characteristics, *C. indica* has a short and dense plant structure with dark green leaves, while *C. sativa* is a tall and skinny plant with pale green appearance.^14,15^

The *C. sativa* plants are also categorized into industrial hemp plants and medicinal plants. Hemp plants are grown for fiber and seed oil production, animal feed, and for their high nutritional value.^6^ Both plants contain the THC, but medicinal plants generally have a higher content of THC that is concentrated in the plant resin glands.^16–18^ Hemp plants are known to contain low resin levels, while medical plants are high in resin levels that cover the female's flowering tops and leaves. The THC concentration in flowering tops is significantly more than other parts of the plant.^16,17,19^ In addition, studies reveal that the THC:CBD ratio in *C. sativa* is generally higher than *C. indica*. Therefore, the euphoric and relaxing effects, after using *C. sativa*, are likely to be greater than with *C. indica*.^15,16^

### Cannabinoid receptors

The bodies' endogenous opioid and cannabinoid systems have remained unchanged for more than 500 million years of human evaluation.^17^ The endogenous cannabinoid system contains endocannabinoids, cannabinoid receptors, second messengers, and anabolic and catabolic enzymes such as the fatty acid amide hydrolase (FAAH) system.^17,20,21^ Cannabinoid receptors located on cell membranes are members of the G-protein-coupled receptor (GPCR) family.^22,23^ Cannabinoid receptor-1 (CB1) is found in moderate levels mainly in the brain and neural tissues and in lower levels in respiratory, digestive, and reproductive systems and urinary tracts.^22,24^ It is mainly responsible for the psychological impacts of THC.^18,25^

Cannabinoid receptor-2 (CB2) is mainly found in peripheral organs, specifically rich in thymus, tonsils, and spleen of immune system.^9,26,27^ CB2 sequence appears more preserved between species than CB1 receptor. Furthermore, there is a third putative human cannabinoid receptor, G-protein-coupled receptor 55 (GPR55).^21^ Studies have shown that malignant cells can alter the physiologic function of GPR55 and other GPCRs to enhance tumor growth and metastasis.^28,29^ The endocannabinoids, 2-AG and AEA, are among the main endogenous cannabinoids that stimulate CB1, CB2, and GPR55, and also act on transient receptor potential ion channels, including transient receptor potential vanilloid (TRPV).^20,21,24^

There are emerging data on other potential cannabinoid receptors, but to date, CB1, CB2, and GPR55 are considered the main cannabinoid targets.^30^

### THC and CBD and their effects on cancer

THC is responsible for most of the psychological effects of cannabis.^31^ THC was first discovered to inhibit the growth of lung adenocarcinoma cells *in vitro* and *in vivo*.^5^ THC binds to activate cannabinoid receptors, predominantly CB1 contained at high concentrations in specific brain regions associated with emotions, thinking, perception, memory, and coordination.^32^ THC works like the AEA neurotransmitter that is produced in the body to regulate perception of pain, eating, and sleeping habits. Except for the initial relaxed state, it can bring about anxiety, delusions, hallucinations, and emotions of happiness. Its effects begin immediately or 30 min after consumption, depending on the route of administration.^33^

CBD refers to that main cannabis compound with desirable medical characteristics, without the stoning effects.^31^ The discovery of CBD was in 1940, which is more than 20 years earlier than THC.^34^ It simulates cannabinoid (CB1 and CB2) receptors indirectly,^35^ and also involves several other receptor pathways that include but are not limited to TRPV1, GPR55, and peroxisome proliferator-activated receptors.^21,36^ CBD in particular increases the susceptibility of tumor cells to the lymphokine-activated killer cells, which can result in cell lysis.^37^

Cannabis rich in CBD is less psychoactive than those rich in THC. Dominant CBD strains are more suitable for applications in relieving spasms and pain, psychosis, anxiety, inflammation, and some other conditions, with lesser effects on lethargy and lower likelihood of causing dysphoria.^38^ It can be of value in treating depression and some side effects that result from cancer treatment.^39^ Overall, CBD is preferred for medical applications and has fewer health risks compared with THC and is useful in negating some of the adverse effects of THC.^36,40^

### Mechanism of action

*In vitro* models for different cancer types have been utilized to elucidate mechanisms by which these cannabinoids and endocannabinoids influence the proliferation, migration, and apoptosis of cancer cells.^5^ The mechanisms to affect cancer cell proliferation, migration, and apoptosis by cannabinoids are complex and can differ between cancer types.^41–43^ They can also inhibit tumor vascularization by altering the morphology of blood vessels and reducing blood vessel proangiogenic factors, including vascular endothelial growth factor.^42,43^

Cannabinoids stimulate the production of ceramides by activation of ceramide synthase enzyme,^44^ which causes a downstream activation of a cascade that signals through the extracellular regulated kinase (ERK) system leading to cell cycle arrest and apoptosis. When CB1 and CB2 receptors are activated, the stress-regulated protein p8 controls the activating transcription factor 4 (ATF-4), C/EBP-homologous protein (CHOP), and tribbles homologue3 (TRB3) expression. The ceramide-ERK signaling pathway is triggered to promote mitochondrial intrinsic apoptosis via mechanisms that have not yet been recognized.^45^ An increase in ceramide can also activate the p38 mitogen-activated protein kinase pathway leading to apoptosis through several mechanisms.^10,42,46^

Activation of CB1 or CB2 receptors can also inhibit the activity of adenylyl cyclase and decreases the levels of cyclic adenosine monophosphate^47^ and the activation of protein kinase A. As a result, there is a decrease in gene transcription causing apoptosis.^41^ Activation of cannabinoid receptors can also promote cancer cell survival and inhibit apoptosis through PI3K/PKB pathways, implicated in cell survival.^48^

### Effects of cannabinoids on pancreatic cancer

#### Effects of cannabis receptors

Studies showed that CB1 and CB2 receptors are expressed in pancreatic cancer cells and have very low or nondetectable mRNA levels in normal pancreatic cells.^49^ In a study by Michalski et al.,^49^ the effects of cannabinoids in pancreatic cells were investigated, with identification of cannabinoid receptor expression in several human pancreatic cancer cell lines and human pancreatic cancer biopsies. These results indicated that activation of cannabinoid receptors, particularly CB2, may induce pancreatic cancer cell apoptosis without affecting the normal pancreas cells.

Michalski et al. also evaluated cannabinoid receptors together with the endocannabinoid metabolizing FAAH and monoacylglycerol lipase (MGLL) enzymes in both normal (n:10) and pancreatic cancer cells from patients with resected pancreatic ductal adenocarcinoma (n:40). Immunohistochemistry staining showed that immunoreactivity for FAAH and MGLL was low in healthy human pancreatic cells, as well as islets and nerves when compared with the immunoactivity for FAAH and MGLL in pancreatic cancer tissues, although low levels of these enzymes in pancreatic cancer cells were correlated with shorter survival.

Wide intrapancreatic nerves were found to be immunoreactive for FAAH and MGLL. In addition, there was a correlation between low CB1 receptor expression and prolonged survival for patients with pancreatic cancer. CB2 receptor expression did not correlate with survival. The levels of endocannabinoids AEA and 2-AG in serum of patients with pancreatic cancer cells in comparison with normal controls were similar.^49^

#### *In vitro* studies

Cytotoxic effects of cannabis *in vitro* occur through cannabinoid receptor-dependent and cannabinoid receptor-independent mechanisms.^50,51^ A study by Fogli et al. showed that cannabis receptor binding by synthetic receptor agonists, WIN-55,212-2 (CB1 and CB2), ACEA (CB1), and JWH-015 (CB2), caused a substantial cell death of MiaPaCa-2 cell.^52^ The authors also demonstrated that the CB1 inverse agonist (AM251) induced apoptosis and affected transcriptional genes via the JAK/STAT and MAPK signaling network in MiaPaCa2 pancreatic cancer cells, acting through CB1 receptor-independent pathways.^52^ In addition, AM251 synergically increased the anticancer effect of pyrimidine analog chemotherapy agent, 5-fluorouracil.^52^

It is possible, however, that these agents have CB1 agonistic effects at higher concentrations, in keeping with findings reported for a structurally similar ligand, SR141716A.^53,54^

The CB1 and CB2 selective agonists, arachidonylcyclopropylamide (ACPA) and GW, inhibited proliferation and invasion of Panc1 cells, respectively.^55^ ACPA and GW through activating the CB1 and CB2 receptors stimulated 5' adenosine monophosphate-activated protein kinase activation via a reactive oxygen species (ROS)-dependent escalate of AMP/ATP ratio, causing cell autophagy and cell growth inhibition.^55,56^

Ceramide synthesized by *de novo* synthesis was found to be involved in the apoptosis of pancreatic cancer cells induced by THC.^57^ THC has been shown to cause a dose-dependent reduction in cell viability by inducing apoptotic cell death.^58^ The stress-regulated protein p8 was involved in the apoptosis of pancreatic cancer cells caused by THC. This protein has been shown to increase with ceramide treatment and has powerful antitumor effects.^59^ There was an increase in p8 mRNA levels in MiaPaca2 pancreatic cancer cells treated with THC, which was blocked by a potent antagonist of CB2 (SR144528). Knockdown of p8 mRNA prevented apoptosis induced by THC in MiaPaCa2 cells.^59^

Furthermore, several p8-dependent genes, including the endoplasmic reticulum (ER) stress-related gene TRB3 (a novel ER stress-inducible gene), CHOP, and ATF-4, were identified and associated with apoptotic signaling. The mRNAs of TRB3, CHOP, and ATF-4 were increased to a similar degree as p8 by THC treatment.^45^ Moreover, removing the p8 mRNA prevented upregulation of TRB3, CHOP, and ATF-4. THC induced a p8- and ceramide-mediated apoptotic response in pancreatic cancer cells through upregulation of these genes.^45^

The combination of gemcitabine and cannabinoid receptor agonists in the treatment of pancreatic cancer enhanced intracellular production of ROS, resulting in antiproliferative effects. Gemcitabine stimulated the mRNAs of both CB1 and CB2 via an NF-κB-mediated mechanism.^60^ Nuclear factor-kappaB (NF-κB) inhibitors, MG12 and BAY, blocked gemcitabine-stimulated increase of either CB1 or CB2 mRNAs, while interleukin (IL)-1, an inducer of NF-κB, increased the expression of CB1 and CB2.^60^ Gemcitabine also plays a part in cannabinoid-induced ER stress and antiproliferation.^60^ Cannabinoid-induced autophagy was enhanced by gemcitabine through ROS-mediated mechanism.^60^ In addition, cannabinoids substantially increase the apoptotic effect of gemcitabine.^60^

A recent study showed that the GPR55 receptor, regulated by tumor suppressor p53, may have a crucial role in pancreatic cancer development, via cell cycle regulation and MAPK signaling pathways.^61^ CBD binds to the GPR55 receptor and blocks its activity. Cell growth, cell cycle progression, and MAPK signaling in ASPC1, HPAFII, BXPC3, and PANC1 pancreatic cancer cell lines were inhibited by treatment with the GPR55 antagonist CID16020046 (CID) and CBD. Moreover, HPAFII and PANC1 cell growth was reduced to a greater extent with the combination of CBD and gemcitabine, compared with either treatment alone.^61^ This study demonstrated that the gemcitabine effects on pancreatic cancer cells might be potentiated by inhibition of GPR55.

#### *In vivo* studies

Pre-clinical studies on various cancers have shown that synthetic exogenous, endo-, and phytocannabinoids decrease angiogenesis, growth, and tumor cell migration and induction of apoptosis in cancer cells.^9,62^ The antitumor effects of cannabinoids were also investigated in *in vivo* models of pancreatic cancer. Xenografted MiaPaca2 pancreatic tumor growth was inhibited by THC (15 mg/kg/day) treatment.^58^ The pancreatic tumor growth in an orthotropic model was also inhibited by the synthetic cannabinoid receptor agonist WIN55212,2.^49^ WIN55212,2 also induced apoptosis in pancreatic cancer cells through possibly the activation of TRB3, which is a proapoptotic protein responsible for the apoptosis induced by ER stress.

WIN55212,2 increased the expression of downstream ER stress-related targets involved with apoptosis in pancreatic cancer but not in healthy pancreatic cells, indicating that cannabinoid stimulates apoptosis selectively in pancreatic cancer cells.^58^

The route of cannabinoid administration has significant effects on pharmacokinetics, bioactivity, bioavailability, and effectiveness of drugs. Cannabinoids are water insoluble and cannot be administered intravenously. In addition, cannabinoids are partially degraded by the stomach acids; as a result, oral administration may not be the most effective route for cancer treatment.^63^ The inhalation route of cannabinoids combined with nanomaterials may be useful for targeting lung cancer, but the effectiveness in treating other sites is less certain.^64^ Intratumor (IT) administration of low doses of cannabinoids enhances the effectiveness of drug.^4,65,66^

A study by Yasmin-Karim et al. revealed that a superior pancreatic cancer treatment outcome could be achieved using the combination of cannabinoids and radiotherapy.^7^ Similar findings have been demonstrated in a brain glioma cancer model.^67^ Improved survival was also demonstrated with IT administration of cannabinoids into subcutaneously implanted murine PANC-02 pancreatic cancers measuring 6 mm in maximum dimension.^7^ Furthermore, treating Kras, P53, and Cre mice with a combination of CBD (100 mg/kg) by daily intraperitoneal (IP) administration and gemcitabine (100 mg/kg) by IP administration every 3 days, IP after 80 days of age prolonged the animal survival three times more than vehicle or gemcitabine treatment alone.^61^

Similar findings were shown in a study by Donadelli et al., in which a CB1 binding ligand SR141716 (SR1) combined with gemcitabine reduced tumor growth greater than either agent alone.^60^ Based on their *in vitro* finding, the authors found that an increase in ROS and autophagy pathways may explain the observed synergistic effects *in vivo*.^60^ SR1 has been utilized as an antiobesity agent and is considered a reverse agonist that possessed agonist activity at higher concentrations.^53^ The use of SR1 as an anticancer agent in the clinical situation is, however, unknown, as the drug has been withdrawn from the worldwide market due to unacceptable psychiatric side effects.

### Effects of cannabis on pancreatic stellate cells

Pancreatic stroma contains pancreatic stellate cells (PSCs), immune cells, neural elements, extracellular matrix, and blood vessels.^68^ Interaction of PSCs and cancer cells enhances tumor invasion, growth, chemoresistance, and immunosuppression.^69^ PSCs migrate to damaged sites and induce differentiation and tissue repair promoting fibrosis.^70^ Activation of cannabinoid receptors using WIN55212,2 reduces the migration of PSCs derived from chronic pancreatitis.^70^ This reduction in migration could be related to a reduction production of extracellular matrix and inflammatory cytokines, including IL-6, MCP-1, fibronectin, collagen 1, and α-smooth muscle actin.^70,71^

The reduction of PSC migration by activation of cannabinoid receptors was followed by a reduction in matrix metalloproteinase-2 (MMP-2) levels.^70,72^ Increased MMP-2 level enables deposition of pathogenic fibrillar collagens. Therefore, suppression of MMP-2 by cannabinoids could contribute to reducing collagen synthesis and reducing the inflammation and fibrosis leading to chronic pancreatitis.^70^ Previous studies have demonstrated the positive association between chronic pancreatitis and pancreatic cancer,^73^ and it is possible that administration of cannabinoids may prevent progression from chronic pancreatitis to pancreatic cancer.

## Conclusion

Endogenous cannabinoids, synthetic or cannabis extracted from plants, can reduce tumor invasion and growth, induce tumor cell death, and inhibit tumor angiogenesis via cannabinoid receptor or receptor-independent pathways. Cannabinoid receptors appear to be highly expressed in pancreatic cancer compared with normal pancreatic tissue. CBD and THC appear to have antiproliferative and proapoptotic effects. CBD in a clinically relevant pancreatic cancer model improved survival outcomes when combined with gemcitabine. Clinical studies on the utility of cannabinoids in the treatment of pancreatic cancer are lacking and urgently needed.

## Abbreviations Used

2-AG: 2-arachidonoylglycerol
AEA: anandamide
ATF-4: activating transcription factor 4
CB1: cannabinoid receptor-1
CB2: cannabinoid receptor-2
CBD: cannabidiol
CBN: cannabinol
CHOP: C/EBP-homologous protein
ER: endoplasmic reticulum
ERK: extracellular regulated kinase
FAAH: fatty acid amide hydrolase
GPCR: G-protein-coupled receptor
GPR55: G-protein-coupled receptor 55
IT: intratumor
MGLL: monoacylglycerol lipase
MMP-2: matrix metalloproteinase-2
NF-κB: nuclear factor-kappaB
PSC: pancreatic stellate cell
ROS: reactive oxygen species
THC: tetrahydrocannabinol
TRB3: tribbles homologue3
TRPV: transient receptor potential vanilloid
